# Atripla^R^/anti-TB combination in TB/HIV patients. Drug in focus

**DOI:** 10.1186/1756-0500-4-511

**Published:** 2011-11-24

**Authors:** Hadija H Semvua, Gibson S Kibiki

**Affiliations:** 1 Kilimanjaro Clinical Research Institute (KCRI), Kilimanjaro Christian Medical Centre (KCMC), P.O Box 2236, Moshi-Kilimanjaro, Tanzania

**Keywords:** Tuberculosis, HIV, Emtricitabine, Tenofovir, Efavirenz, Interaction

## Abstract

**Background:**

Co-administration of anti-tuberculosis and antiretroviral therapy is often inevitable in high-burden countries where tuberculosis is the most common opportunistic infection associated with HIV/AIDS. Concurrent use of rifampicin and several antiretroviral drugs is complicated by pharmacokinetic drug-drug interaction.

**Method:**

Pubmed and Google search following the key words tuberculosis, HIV, emtricitabine, tenofovir efavirenz, interaction were used to find relevant information on each drug of the fixed dose combination Atripla^R^

**Results:**

Information on generic name, trade name, pharmacokinetic parameter, metabolism and the pharmacokinetic interaction with Anti-TB drugs of emtricitabine, tenofovir, and efavirenz was obtained.

**Conclusion:**

Fixed dose combination of emtricitabine/tenofovir/efavirenz (ATRIPLA^R^) which has been approved by Food and Drug Administration shows promising results as far as safety and efficacy is concerned in TB/HIV co-infection patients, hence can be considered effective and safe antiretroviral drug in TB/HIV management for adult and children above 3 years of age.

## Background

Human immunodeficiency virus (HIV) and tuberculosis (TB) are overlapping epidemics that cause an immense burden of disease. Sub-Sahara Africa is a region most affected by both diseases including Tanzania [1]. Literatures show that more than 75% of TB patients have also HIV, and possibly more than half of worldwide patients infected with HIV will also develop TB [2,3]. Furthermore TB contributed to 27% of all AIDS diagnoses [4]. For TB the currently used combination drug regimens produce cure rates that exceed 95%, given good patient adherence during the multiple months' treatment period [5]. Also for HIV many regimens are available; however optimal treatment regimens for TB/HIV co-infection are not yet clearly defined.

As therapy for HIV disease becomes more available, physicians need to know how to treat these two diseases effectively while minimizing the risk of drug interactions and maintaining the shortest possible duration of treatment for TB. Current treatment of mycobacterium tuberculosis in most resource limited settings is comprised of a four-drug initial anti-tuberculosis regimen for 2 months (rifampicin, isoniazid, pyrazinamide and ethambutol), followed by two-drugs continuation phase of anti-tuberculosis regimen for 4 months (rifampicin and isoniazid). For TB/HIV co-infected patients the guidelines which exist [6] have shown many challenges.

Combining drug therapies for dual infection TB and HIV is made complex by alterations in the activity of the hepatic Cytochrome P450 (CYP) system, high pill burdens, shared drug toxicities, drug-drug and drug-disease interactions, immune reconstitution inflammatory syndrome, co-morbid diseases and drug resistance in both bacillus and virus [7,8]. The CYP isoform enzymes are responsible for many interactions [9] (especially those involving rifampicin and isoniazid) during drug biotransformation (metabolism) in the liver and/or intestine, due to it is enzyme induction effect.

### Presentation of the hypothesis

Adherence to a complex regimen is often a significant barrier to treatment success. Following the current guideline where a patient has to take 4-fixed dose combination for TB and the complex triple combination therapy selected for HIV treatment. The consequence of many pills reduce compliance and hence adherence to the treatment regimen leading to suboptimal TB and HIV treatment, hence increasing the possibility of drug resistance and shared drug toxicities [10]. These toxicities may necessitate therapy discontinuation, which exacerbates immune suppression and predisposes to other opportunistic infections.

TB/HIV co-infection simultaneous treatment have shown pharmacokinetic interactions produced with mainly rifampicin [(a cornerstone in TB treatment) and the non nucleoside reverse transcriptase inhibitors (NNRTIs)]. Enzyme inducer decreases elimination halve life of nevirapine [11]. When rifampicin and nevirapine are given together there is an observed 31% to 58% decrease in plasma levels of nevirapine due to rifampicin induction effect [12-15]. Available literatures explain that rifampicin combination with efavirenz there is reduction of serum levels of 13-33% [16], however no virological failure reported to be significant [17]. HIV-infected patients achieve somewhat lower concentrations of the orally administered first-line anti-tubercular drugs [18,19]. The effect of rifampicin on the concentrations of Protease Inhibitors (PI) is well elaborated in a study of Moreno et al. [20].

Immune reconstitution inflammatory syndrome (IRIS), which is due to dysregulated immune recovery [21], occurs in severely immune-suppressed HIV patients normally 1 to 4 weeks after ART initiation [22,23]. In tuberculosis-related IRIS which is an inflammatory reaction is directed towards mycobacterial antigens [24], resulting in worsening pulmonary infiltrates. Risk factors for TB-IRIS include low baseline CD4 count, high baseline viral load, short duration between TB and ART initiation and disseminated tuberculosis [22,25], making difficult to determine the optimal time to initiate ART in severely immune-suppressed TB patients [26,27].

Immune reconstitution is based on the patient's ability to respond to treatment. For the most part, in patients with CD4 counts of 200-350 cells/mm^3 ^who start treatment, their immune systems still have the ability to be activated to produce more CD4 cells [28]. In contrast, in patients with low CD4 counts (< 100 cells/mm^3^), it may be more difficult to activate their immune systems to produce many CD4 cells because of existing damage from the virus.

In the management of TB/HIV co-infection directly observed therapy and other adherence promoting strategies should be used in all patients with HIV-related TB [29]. Whenever possible, the care for HIV-related TB should be provided by or in consultation with experts in management of both TB and HIV. The care for persons with HIV-related TB should include close attention to the possibility of TB treatment failure, antiretroviral treatment failure, paradoxical reactions, side effects for all drugs used, and drug toxicities associated with increased serum concentrations of rifampicin.

Due to these accompanied complications, selecting an appropriate antiretroviral therapeutic regimen is warranted. This involves addressing multiple interdependent issues, including patient adherence, pharmacokinetic properties of the drugs (including food effects and drug-drug interactions), drug resistance, and overlapping adverse effects. On 12 July 2006 Bristol-Myers Squibb and Gilead Sciences announced that the US Food and Drug Administration (FDA) had cleared Atripla^R^, their fixed-dose combination tablet. Atripla^R ^is a complete regimen in a single, fixed-dose combination tablet that contains: efavirenz 600 mg, emtricitabine 200 mg and tenofovir disoproxil fumarate 300 mg. It is a novel co formulation drugs from two different classes, simplifying administration and increasing adherence too [30].

### Testing the hypothesis

A new formulation combining fixed doses of the nucleoside reverse transcriptase inhibitors emtricitabine (200 mg) and tenofovir disoproxil fumarate (tenofovir DF; 300 mg) with the non-nucleoside reverse transcriptase inhibitor efavirenz (600 mg) represents the first once-daily, one-tablet antiretroviral regimen. Co-formulated efavirenz/emtricitabine/tenofovir DF demonstrated excellent potency, tolerability and favorable safety profile [31].

Co administration of co-formulated efavirenz/emtricitabine/tenofovir DF with rifampicin based TB regimen clinical trials is going on in TB/HIV patients of Tanzania, therefore is important to have knowledge of each individual drug. Also a once-daily regimen of efavirenz, emtricitabine and tenofovir DF (administered as individual agents) was superior to once-daily efavirenz plus twice-daily co-formulated lamivudine/zidovudine in terms of virological suppression, immunological recovery and adverse events [32]. Individually, these agents have long half-life that allow for once-daily dosing.

**Emtricitabine **emtriva^R ^a cytosine analogue is the newest of the nucleoside reverse transcriptase inhibitor (NRTI) drugs. It was well tolerated in clinical trials where most adverse events were consistent with the NRTI class. Moreover, emtricitabine based regimens were as well tolerated as those with lamivudine [33]. Emtricitabine has no currently known phase I (glucuronidation) or phase II (cytochrome P450 and others) interactions [34]. It may be taken with or without food [35]. Emtricitabine displays dose-proportional pharmacokinetics. Bioavailability is not affected by food intake. Mean steady-state C_max_, T_max _and AUC values were 1.7 mg/L, 2 h, and 10 mg/L.h respectively in 6 patients with HIV infection. Plasma elimination half-life is 8-10 h, but the intracellular half-life of the active compound, emtricitabine-triphosphate is much longer: 39 h which support the once daily dosing.

**Tenofovir disoproxil fumarate **Viread^R ^is an analogue of adenosine five monophosphate. It is a nucleotide analogue and consequently its mechanism of action differs from that of nucleoside analogues. It is a prodrug, therefore after absorption the drug is hydrolyzed and the active compound tenofovir-diphosphate is released. It has a longer serum half-life than most other NRTIs allowing a once daily dosing. The plasma half-life is 14.4 h, but the intracellular elimination half-life of the active compound, tenofovir-diphosphate is much longer > 49 h.

This drug has broad tissue distribution, aided by its small molecular size and very low protein binding, and is eliminated as unchanged drug in the urine through glomerular filtration and active tubular secretion. Because of this latter characteristic, dosage adjustments are required in patients with renal insufficiency [36]. 70-80% of a dose is recovered unchanged in the urine, suggesting minimal influence of hepatic metabolism. Tenofovir mean steady-state C_max_, T_max _and AUC were 0.33 mg/L, 2.3 h, and 3.0 mg/L.h respectively in patients infected with HIV. Adverse reactions are relatively uncommon, and nausea is the most often reported symptom [37]. The bioavailability of tenofovir disoproxil fumarate is increased in the presence of a high fat diet (40%-50%), and the drug should be taken with food, [38,39]

Tenofovir disoproxil fumarate is not a substrate, inhibitor, or inducer of the P450 system, within the class of antiretroviral agents, an increase in the bioavailability of didanosine has been described, leading to the recommendation that the dose of didanosine be reduced when used in combination with tenofovir. Tenofovir can be used without adjustments with other nucleoside and nonnucleoside reverse transcriptase inhibitors. Equally, tenofovir seems to have no effect on the pharmacokinetics of protease inhibitors although these latter agents may produce a slight increase in the bioavailability of tenofovir, which seems to be of little clinical relevance.

**Efavirenz Sustiva^R ^**is a NNRTI which is principally metabolized by cytochrome P450, more precisely isoenzymes CYP2B6 and CYP3A4. No active metabolites are formed. Efavirenz has a long terminal half-life of 40-55 h after multiple dosing. Neuropsychiatric symptoms are the common side effects observed. Fumaz et al. [40], in a randomized, prospective, two-arm controlled study compared quality of life and neuropsychiatric side effects in patients receiving an efavirenz-containing regimen versus a group whose treatment did not include efavirenz. They found that the group receiving an efavirenz-containing regimen reported a better quality of life, particularly because their regimen was easier. They found, however, that 13% of their patients reported "character change". Meals of normal composition have no appreciable effect on the pharmacokinetics of efavirenz. The bioavailability is increased following a high fat meal. Time to peak plasma concentration is 3-5 h. Steady state concentrations are achieved after 6-7 days. Mean steady state C_max_, C_min _and AUC_(0-24 h) _were 4.1 mg/L, 1.8 mg/L and 58.1 h*mg/L respectively after multiple dosing of 600 mg efavirenz once daily.

Several pharmacokinetic studies have been conducted showing the interaction of rifampicin with efavirenz. Lopez-Cortez et al. [16] in Spain did on 24 patients with concomitant infections of HIV and tuberculosis. It was nonblinded study where group A (*n *= 16) received antituberculosis drugs without rifampicin but with added efavirenz 600 mg/day ARV regimen. The patients were then switched to a standard antituberculosis regimen containing rifampicin with efavirenz 600 mg/day (group A1; *n *= 8) or efavirenz 800 mg/day (group A2; *n *= 8). The result showed that levels of AUC, Cmax, and Cmin of efavirenz decreased respectively by 24%, 25%, and 22%, after addition of rifampicin to the efavirenz of 600 mg/day group. Significant association of body weight, dose of efavirenz and its pharmacokinetic parameters was observed and the dose increase to 800 mg/day when efavirenz is used with rifampicin was recommended.

Manosuthi et al. [17,41] conducted a non-blinded randomized clinical trial to Thai patients and they were given rifampicin for more than 1 month with regimen containing efavirenz 600 mg/day (*n *= 42), or efavirenz 800 mg/day (*n *= 42). The plasma concentrations of efavirenz were determined for both groups 12 h after drug administration and on day 14. Both groups had 12-h efavirenz concentrations (C_12_) of same value (median 3.0 mg/L vs 3.3 mg/L); but the upper C_12 _levels were higher in the group receiving efavirenz at the dosage of 800 mg/day (21.3 mg/L) in comparison with the group on 600 mg/day (12.2 mg/L). The study concluded that efavirenz with a dose of 600 mg/day is adequate for the majority of HIV-infected patients in Thailand (as their body weight is around 50 kg) but more studied should be performed to other ethnic groups. This is in line with a recent study by Orrell [42] which showed that 600 mg dose of efavirenz is adequate.

Another study was conducted aiming at determining the effects of higher body weights and differing ethnicities on exposure to efavirenz. 9 patients with body weights of over 50 kg were enrolled in the study [43]. The patients received an antiretroviral regimen containing efavirenz 800 mg/day and antituberculosis therapy containing rifampicin. Plasma concentrations of efavirenz were monitored for 2 years. All patients had sub-therapeutic trough concentrations of efavirenz (median 11.7 mg/L; range 5.37-19.6 mg/L). The expected therapeutic range of trough concentrations of efavirenz were between 1.2 and 4.0 mg/L. Seven out of the 9 patients with HIV/TB co-infection had toxicity (6 of them black and 1 caucasian). Around 20% of ethnic Black Africans possess homologous G516T alleles versus 3% of Caucasians [44]. This CYP2B6 polymorphism is associated with exposure to higher concentrations of efavirenz.

Existence of a wide variability in efavirenz concentrations among black Africans was observed in the study of Friedland et al. [45]. This study indicated that inter-subject variability in efavirenz concentrations was greater when the drug was administered with rifampicin (coefficient of variation [CV] 157%) compared with the inter-subject variability after discontinuation of rifampicin (58%), with much of consistent intra-subject variation over time (CV 24%). However, Cmin was not significantly different during versus post TB therapy.

Regarding metabolism studies have shown that CYP2B6 genetic polymorphisms influence very much efavirenz elimination [46-48]. CYP2B6 polymorphisms have been associated with altered PK of efavirenz in HIV-infected adults and children [49-51] resulting in a higher efavirenz exposure and an increase in central nervous system side effect [44,46]. CYP2B6 patients are poor metabolizer of the drug and dose reduction has been proposed to people with this genotype [52,53]. Of note: is the high prevalence of efavirenz related CYP2B6 polymorphisms in patients of African region which represent the majority of the HIV-infected population in the world [54-58]. This indicates the need for prospective clinical studies to evaluate the utility of dose adjustments in these populations.

### Use of efavirenz in children

The use of efavirenz as a component of first-line antiretroviral therapy in adult and children above 3 years has been accepted worldwide [59], however safety and efficacy of efavirenz in children less than 3 years of age has not been performed [60-63] and the data on the efavirenz exposure in mothers and their breastfed infants is also limited [64]

Despite the report that efavirenz is highly effective and well tolerated in the majority of pediatric patients [65], efavirenz plasma concentrations have been reported to be suboptimal in a large proportion of children and adolescents dosed according to current pediatric guidelines [60,61] Also interindividual variability has been observed in paediatric population [66].

Recently data on population pharmacokinetics (PK) of efavirenz in children predicts sub-therapeutic efavirenz exposure in a significant proportion on children with the currently recommended efavirenz dose [61,67]. Authors in ARROW study which was done recently in Uganda and Zimbabwe [68] suggested the use of higher paediatric efavirenz doses, as per WHO 2010 recommendations, however they concluded that children with toxic level of efavirenz concentration may be increased. Also efavirenz pharmacokinetics was shown to demonstrate significant age effects with apparent clearance much greater in young infants than older children. High efavirenz dose requirements and pharmacokinetic variability in infants will require additional pharmacokinetic studies to determine appropriate efavirenz dosing strategies. Therefore priorities should be made to access the developmental changes of efavirenz exposure in children and adolescents and application of therapeutic drug monitoring to the settings where the facility is available.

### Implication of the hypothesis

HAART regimens have contained very high pill burdens, complex dosing schedules, and specific food requirements. The management of HIV-related tuberculosis disease seems to be complex although the treatment of TB in persons with HIV is essentially the same as for patients without HIV. A major concern in treating TB in HIV-infected persons is the interaction of rifampin with certain antiretroviral agents, PIs and NNRTIs [69,70]. Rifabutin, which has fewer drug interactions, may be used as an alternative to rifampicin but is expensive and not available in resource limited area.

Pharmacokinetic data which are present indicate that rifampicin can be combined with efavirenz [16,17].or nevirapine,[14] although for TB/HIV co-infected patients who got 600 mg rifampicin, adverse events were reported to be higher in nevirapine based regimen compared to efavirenz based regimen [71]. Non-boosted PIs cannot be administered with rifampicin because of considerable decreases in plasma concentrations of PIs, which may cause sub-therapeutic concentrations except ritonavir[72,73]. Ritonavir is a strong inhibitor of CYP3A4 and P-glycoprotein, and when it is low dose is combined with other PIs an important increase in their plasma concentrations is achieved, however this is not a solution as substantial decreases in plasma concentrations of indinavir,[74] lopinavir [75] and atazanavir [76] have been observed when co-administered with rifampicin, even when small doses of ritonavir are given. Another promising PI is saquinavir although in a study of Ribera et al. [77] a decrease of 40%, 35% and 49% in the median saquinavir AUC_0-24_, Cmax and Ctrough, respectively, were observed.

In vitro study reveals the benefit from the use of combination therapy achieved with Atripla in terms of the emtricitabine and tenofovir intracellular drug concentration [78]. However in this study patients were HIV positive only hence more data are needed from TB/HIV co-infected patient.

HIV fusion inhibitors represent a novel class of antiretroviral drugs and enfuvirtide is the first drug within this class to be approved [79]. When combined with active ARV drugs, different studies reported findings of substantial improvements in virological, immunological and clinical outcomes [80-83]. It has little potential for drug-drug interactions as far as coadministration with TB drugs is concerned [79,84,85]. The major limitation of this drug is the twice daily dosing which may interfere with the treatment adherence. Injection-site reactions occurred almost universally in its recipients, although did not necessitate stopping treatment.

Co-formulated atripla^R ^(emtricitabine/tenofovir/efavirenz) have demonstrated excellent potency, tolerability and favorable safety profile. The drug is now available in most of the limited-resources countries. The drug is given once per day simplifying administration and increasing adherence too, hence may be recommended as a way to jointly treat these two diseases.

## List of abbreviations

ARV: Antiretroviral therapy; CYP: Cytochrome 450 system; FDA: Food and drug administration; HAART: Highly active antiretroviral therapy; HIV: Human immunodeficiency virus; IRIS: Immune reconstitution inflammatory syndrome; NNRTIs: Nonnucleoside reverse transcriptase inhibitors; NRTIs: Nucleoside reverse transcriptase inhibitors; PIs: Protease inhibitors; TB: Tuberculosis.

## Competing interests

The authors declare that they have no competing interests.

## Authors' contributions

GK gave an idea based on the fact that a phase II clinical trial is going on in our institute where TB/HIV co-infected patients are given Atripla^R ^in combination with 4FDC tuberculosis drugs, so knowing the detail of the drug involved is important. HS contributed in writing the manuscript. Both authors read and approved the final manuscript.
